# Prospective prediction of childhood body mass index trajectories using multi-task Gaussian processes

**DOI:** 10.1038/s41366-024-01679-0

**Published:** 2024-11-15

**Authors:** Arthur Leroy, Varsha Gupta, Mya Thway Tint, Delicia Shu Qin Ooi, Fabian Yap, Ngee Lek, Keith M. Godfrey, Yap Seng Chong, Yung Seng Lee, Johan G. Eriksson, Mauricio A. Álvarez, Navin Michael, Dennis Wang

**Affiliations:** 1 Department of Computer Science, The University of Manchester, Manchester, UK; 2 Institute for Human Development and Potential, Agency for Science Technology and Research (A*STAR), Singapore, Republic of Singapore; 3 Bioinformatics Institute, Agency for Science Technology and Research (A*STAR), Singapore, Republic of Singapore; 4 Department of Paediatrics, Yong Loo Lin School of Medicine, National University of Singapore, Singapore, Republic of Singapore; 5 Department of Paediatrics, KK Women’s and Children’s Hospital, Singapore, Republic of Singapore; 6 Duke-NUS Medical School, Singapore, Republic of Singapore; 7 MRC Lifecourse Epidemiology Centre and NIHR Southampton Biomedical Research Centre, University of Southampton and University Hospital Southampton NHS Foundation Trust, Southampton, UK; 8 Department of Obstetrics and Gynaecology and Human Potential Translational Research Programme, Yong Loo Lin School of Medicine, National University of Singapore, Singapore, Republic of Singapore; 9 Division of Paediatric Endocrinology, Department of Paediatrics, Khoo Teck Puat-National University Children’s Medical Institute, National University Hospital, National University Health System, Singapore, Republic of Singapore; 10 Department of General Practice and Primary Health Care, University of Helsinki, Helsinki, Finland; 11 Folkhälsan Research Center, Helsinki, Finland; 12 Department of Computer Science, University of Sheffield, Sheffield, UK; 13 National Heart and Lung Institute, Imperial College London, London, UK

**Keywords:** Obesity, Paediatrics

## Abstract

**Background:**

Body mass index (BMI) trajectories have been used to assess the growth of children with respect to their peers, and to anticipate future obesity and disease risk. While retrospective BMI trajectories have been actively studied, models to prospectively predict continuous BMI trajectories have not been investigated.

**Materials and methods:**

Using longitudinal BMI measurements between birth and age 10 y from a mother-offspring cohort, we leveraged a multi-task Gaussian process approach to develop and evaluate a unified framework for modeling, clustering, and prospective prediction of BMI trajectories. We compared its sensitivity to missing values in the longitudinal follow-up of children, compared its prediction performance to cubic B-spline and multilevel Jenss-Bayley models, and used prospectively predicted BMI trajectories to assess the probability of future BMIs crossing the clinical cutoffs for obesity.

**Results:**

MagmaClust identified 5 distinct patterns of BMI trajectories between 0 to 10 y. The method outperformed both cubic B-spline and multilevel Jenss-Bayley models in the accuracy of retrospective BMI trajectories while being more robust to missing data (up to 90%). It was also better at prospectively forecasting BMI trajectories of children for periods ranging from 2 to 8 years into the future, using historic BMI data. Given BMI data between birth and age 2 years, prediction of overweight/obesity status at age 10 years, as computed from MagmaClust’s predictions exhibited high specificity (0.94), negative predictive value (0.89), and accuracy (0.86). The accuracy, sensitivity, and positive predictive value of predictions increased as BMI data from additional time points were utilized for prediction.

**Conclusion:**

MagmaClust provides a unified, probabilistic, non-parametric framework to model, cluster, and prospectively predict childhood BMI trajectories and overweight/obesity risk. The proposed method offers a convenient tool for clinicians to monitor BMI growth in children, allowing them to prospectively identify children with high predicted overweight/obesity risk and implement timely interventions.

## Introduction

The increasing global prevalence of childhood obesity represents a major concern, given its strong links to comorbidities like cardiometabolic disease as well as psychopathologies [1, 2]. Not all children with obesity have adverse cardiometabolic or mental health [3, 4], and not all children who develop these conditions get obesity. This suggests that there might be aberrant growth patterns even within the normal weight range that increase risks for adverse cardiometabolic or mental health. Periods of accelerated weight gain in childhood can result in atherosclerotic changes and increased body fat percentage, which can persist even if the child reverts to normal weight later in life [5, 6]. Therefore, tracking and characterizing longitudinal childhood growth can provide a more refined picture of the predispositions for adverse health outcomes.

Parents and pediatricians are often interested in the retrospective question of how a child has grown relative to his/her peers. This is often assessed using simplistic approaches like plotting growth assessments on childhood growth charts [7]. While such approaches are useful for detecting aberrant growth trends (e.g., growth faltering/stunting) that require prompt interventions, these provide a crude or misleading picture of how an individual child will grow in the future. While there has been a large body of literature that has focused on retrospective growth assessments using growth clustering [8–12] and individual body mass index (BMI) trajectory modeling [12–17], not many studies have systematically investigated whether continuous BMI trajectories can be prospectively forecasted from prior BMI assessments. Knowing this would be useful for evaluating if/when a child could be expected to cross standard overweight/obesity cut-offs and for prioritizing children for preventive interventions. A child’s BMI trajectory can also reveal more subtle features of growth like growth velocities [18] and alterations in milestones like infancy BMI peak and adiposity rebound, which have been linked to later obesity risk.

In the current work, we propose to use a Gaussian processes (GP) algorithm called MagmaClust [19, 20] for modeling, clustering and prospectively forecasting childhood BMI trajectories. A GP is a random process over functions (or curves) that is characterized by a specific mean and covariance function [21]. GP-based methods offer a probabilistic non-parametric framework by defining a prior distribution over functions, allowing us to capture complex non-linear relationships while accounting for uncertainty. Observed individual BMI trajectories can be thought of as specific instantiations of different GPs.

In this study, we extended MagmaClust to perform multiple tasks of learning by sharing information across individual BMI trajectories while clustering the trajectories into distinct patterns. We show that it can perform uncertainty quantification of BMI trajectories from a cohort of children followed up from ages 0–10 years. The algorithm focuses on predicting probabilities for missing BMI values at time points when individuals were not measured, outperforming existing methods. When given historic BMI measurements, MagmaClust is able to prospectively compute the probability of acquiring overweight/obesity at future ages.

## Materials and methods

### Cohort dataset

Longitudinal height and weight data between birth and age 10 y were available for 1177 children from the Growing Up in Singapore Towards healthy Outcomes (GUSTO) cohort [22]. Precise data availability at each of the 20 timepoints between birth and age 10 y are provided in Table S1. Calibrated weighing scales were used for measuring weight (SECA 334 up to 18 m and SECA 803 weighing scale beyond 18 m). Recumbent length (SECA 210 mobile measuring mat) was used to compute BMI until age 2, while standing height (SECA 213 Portable Stadiometer) was used for computing BMI beyond age 2. The growth data of 1177 children were randomly split into a training set (*N* = 600) and a test set (*N* = 577).

### Model fitting

The training set was used to train the MagmaClust model [19, 20]. The test set was used to calculate the evaluation metrics for different experimental conditions and for reference comparisons of MagmaClust with two legacy growth curve fitting approaches: multilevel Jenss-Bayley [13, 15, 23, 24] and cubic B-splines [14, 16, 17]. Spline modeling was performed using the *smooth.spline* function of the *stat* R package. Jenss-Bayley weight and height models were hierarchically fitted with a non-linear mixed effect model using the *saemix* package in R, which was then used to compute the BMI trajectories. Training and prediction with MagmaClust were performed using the dedicated R package MagmaClustR, where all GP kernels were set to be squared exponentiated (SE). Additional details on the choice of kernel and hyper-parameters can be found in the Supplementary Materials. The estimation of cubic B-splines requires at least 4 observed time points (534 test data), whereas in Jenss Bailey, we used a total of 551 test data with at least 2 observations per individual (out of 577 test data with at least 1 observed timepoint used for MagmaClust).

### BMI prediction

The BMI prediction experiment comprised of two separate tasks: missing data reconstruction and forecasting. Missing data reconstruction corresponds to randomly removing varying percentages of the observed data for each individual and using the remaining points to recover the missing BMI values. For forecasting, we retained all points observed before specific age thresholds (ranging from 2 y to 8 y) to predict BMI for all testing points after this threshold until age 10y. While evaluation metrics are computed only from predicted data points in both cases, graphical illustrations of trajectories are displayed as continuous BMI curves between ages 0 to 10 y. The evaluation of performances was assessed through mean squared errors (MSE) and uncertainty quantification was performed using the Weighted 95% Credible Interval Coverage (WCIC95) metric as described in the Supplementary Materials.

## Results

### Clusters of BMI trajectories in early childhood

**Fig. 1:**
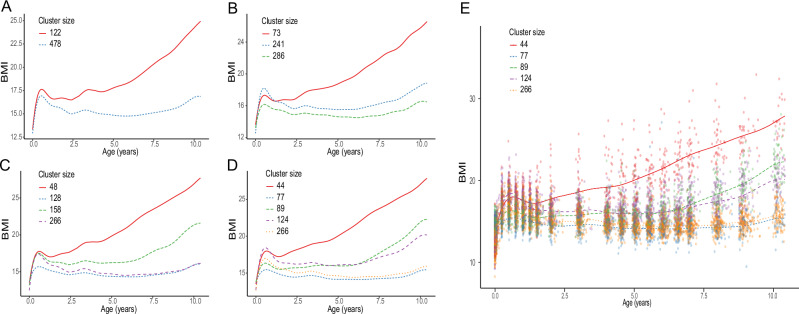
Cluster-specific mean BMI curves with increasing number of clusters. **A** K = 2, (**B**) K = 3, (**C**) K = 4, and (**D**) K = 5. **E** Mean BMI curves associated with K = 5 overlaid on observations from the training data set, colored according to their most probable cluster.

**Fig. 2:**
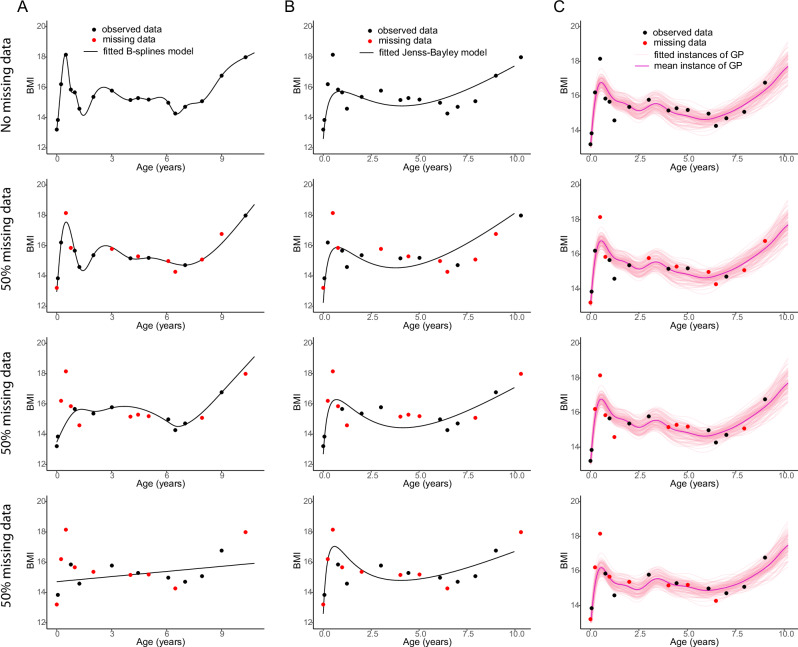
Sensitivity of BMI trajectory models to missing data. **A** B-splines, (**B**) Jenss-Bayley and (**C**) MagmaClust all fit the observed BMI values (black points) well for an individual from birth to age 10. For the same individual, three examples are shown where there is a different set of missing values (red points) occur in 50% of the data. B-splines modeling can lead to large differences in reconstruction, while Jenss-Bayley is more robust. MagmaClust provides robustness, an accurate fit and uncertainty quantification. Note that the slight bump around age 3 y in the B-spline and MagmaClust BMI trajectories are due to the switch from length to height for computation of BMI, and the higher fidelity of these methods to the underlying data trend, when compared to the parametric Jenss-Bayley model.

**Fig. 3:**
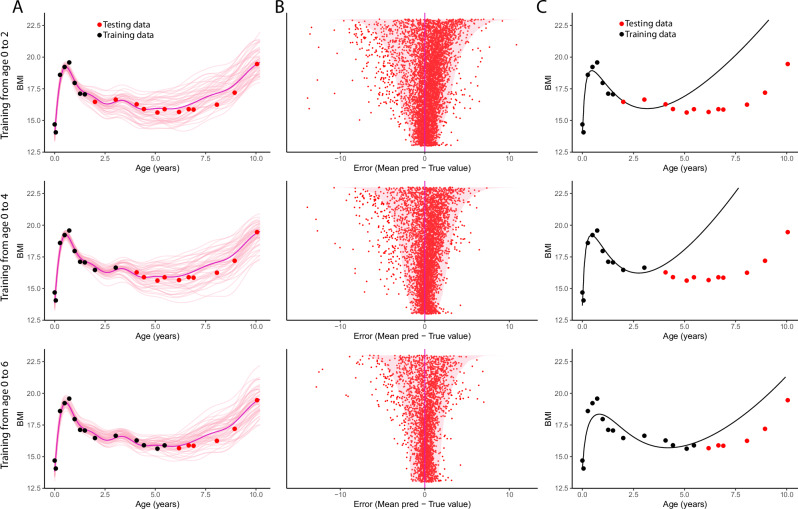
Prospective forecasting of childhood BMI. **A** Illustration of MagmaClust forecasts for a random illustrative individual, observed until age 2 (top), age 4 (middle), and age 6 (bottom). Historic BMI values (black) are used for training the model and the future BMI values (red) are used for evaluating predictions from the model. The thick purple line represents the mean prediction, whereas the thin pink lines correspond to 50 curves sampled from posterior distribution. **B** Uncertainty quantification of errors from MagmaClust’s forecasts across all individuals and all testing points. **C** The Jenss-Bayley model fits a single curve using historic values but its future trajectory deviates from the future BMI values. For all predicted testing points, (sorted by decreasing uncertainty on the *y*-axis), the pink region corresponds to the 95% credible interval predicted from model; and the red dot is the absolute error to the true value.

**Fig. 4:**
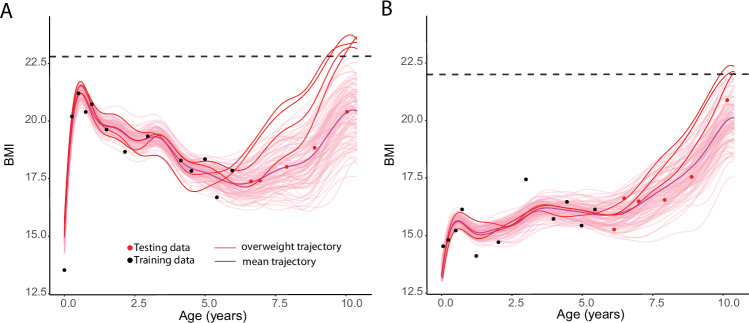
The overweight probability can be estimated from MagmaClust predictions by computing the proportion of posterior instantiations (possible trajectories) crossing the corresponding sex-specific threshold for BMI to be classified as overweight (red lines). **A** Example of a male child showing 4 of the 100 posterior instantiations cross threshold of BMI = 22.8 *kg/m*^2^. **B** Example of a female child showing 3 of the 100 posterior instantiations cross threshold of ${BMI}=22{{Kg}/m}^{2}$.

**Fig. 5:**
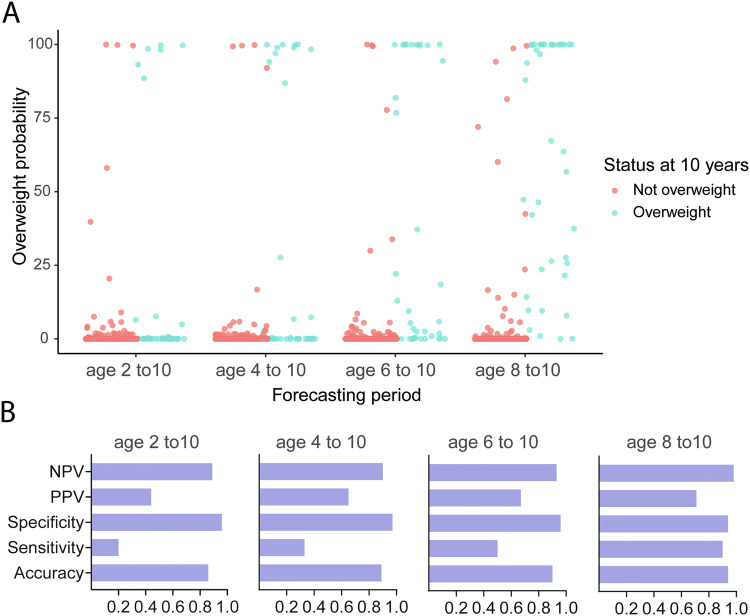
Comparison of observed and predicted overweight status at age 10 y. **A** Visualization of overweight/obesity probabilities estimated from MagmaClust predictions for different forecasting periods. Children are colored according to their observed overweight status at age 10 (overweight threshold is *BMI* > 22 for girls and *BMI* > 22.8 for boys). **B** Measures of negative predictive value (NPV), positive predictive value (PPV), specificity, sensitivity and accuracy for identifying overweight/obesity status at age 10 y for different forecasting ranges (2–10 y, 4–10 y, 6–10 y and 8–10 y) when the overweight/obesity probability decision cutoff was set to 5%.

We applied MagmaClust to longitudinal BMI data collected from children ages 0 to 10 years. The cluster-specific mean BMI trends with increasing number of clusters (K) are illustrated in Fig. 1A–D. We evaluated up to K = 10 clusters. However, for *K* ≥ 6, the additional clusters were empty or contained very few (generally only one) individuals. This suggested that 5 clusters (Fig. 1D) were sufficient to capture the main trends present in the current dataset. When the number of clusters increases from 2 (Fig. 1A) to 5 (Fig. 1D), we observed that while the upper cluster pattern, growing towards BMI values of 26 kg/m^2^ at age 10 y (Fig. 1A), remained roughly similar, the other cluster seemed to split into more specific sub-clusters (Fig. 1B–D). An infant BMI peak (with varying peak intensities) was observed at around 9 months for all clusters, with distinctive BMI patterns after infancy (Fig. 1D). While we observed some of the cluster-specific mean trajectories converged to similar patterns between 3–10 y, they had very distinct trajectories during infancy (age 0–1 y) with different BMI peak levels. Since this is a critical time of child development and there may be important features (eg. different rates of growth acceleration/deceleration) relating to future health outcomes, we chose to retain them as distinct clusters. Figure 1E depicts the mean curves associated with each of the 5 clusters, overlaid on top of the training dataset. Although underlying characteristic patterns can be captured through each cluster’s mean process, note that a continuum of data points exists in between the clusters.

### Comparison of MagmaClust, cubic B-splines and Jenss-Bayley methods for missing data reconstruction

For a randomly chosen individual, we show the BMI trajectories fitted using the MagmaClust, cubic B-splines and Jenss-Bayley using the complete set of BMI measurements in Fig. 2 (1st row) and in different sets of 50% randomly under-sampled BMI measurements in Fig. 2 (2nd to 4th rows). In all rows of Fig. 2A, we observed that the curves fitted with B-splines dramatically vary depending on which specific points are missing. On the other hand, Jenss-Bayley and MagmaClust predictions remain relatively robust regardless of the observed subset (Fig. 2B, C). Regardless of missingness patterns, MagmaClust correctly recovered the mean trend. Uncertainty of predictions (95% credible interval) was shown by displaying multiple trajectories from the posterior distribution (Fig. 2C).

To assess the effect of the number of clusters on the performance of MagmaClust, we randomly removed 50% of the observations in all 577 individuals in the test set and used the remaining observations to compute predictions. The prediction performance with missing data for MagmaClust (from 2 to 5 clusters), Jenss-Bayley and B-splines in Table S2 show lower MSE for MagmaClust, irrespective of the number of clusters. The MSE of the 5-cluster MagmaClust model was, for instance, 24% lower than Jenss-Bayley and 79% lower than B-splines. Overall, the random effects methods (MagmaClust and multi-level Jenss-Bayley) exhibited better performance at missing data reconstruction than those merely relying on individual data (Fig. S1). In Table S3, we compared reconstruction performances of the 5-cluster MagmaClust model with Jenss-Bayley and B-splines when the proportion of missing data increased from 10% to 90%. Overall, MagmaClust’s errors only slightly increased (0.90 to 2.84) when we increased the missing data ratio. Conversely, while B-splines and Jenss-Bayley remained reasonably efficient for low proportions (below 50%), they typically struggled as the missing data ratio increased. Concerningly, the number of computational runtime errors for splines started to rise dramatically above 50% of missing points.

### Comparison of MagmaClust, Cubic B-splines, Jenss-Bayley methods for prospective forecasting of childhood BMI

Using BMI observations collected before different age thresholds (2 y, 3 y, 4 y, 5 y, and 6 y), we predicted the BMI trajectories till age 10 y. Table S4 shows the forecasting performances of MagmaClust, B-splines and Jenss-Bayley models on all testing individuals for a forecasting period going from the age threshold to age 10 y. The Jenss-Bayley model’s MSE was 1.62 times higher than MagmaClust when forecasting from ages 6 to 10 y. This error ratio increased to 3.26 when forecasting was performed using data from ages 2 to 10 y. The performance of splines was much worse, with 9-fold higher MSE for forecasting from ages 6 to 10 y and 86-fold higher MSE for ages 2 to 10 y when compared to MagmaClust. These findings highlight the unsuitability of splines in forecasting tasks, particularly for long-term forecasting. Overall, by identifying relevant patterns from early BMI measurements, MagmaClust demonstrated its ability to forecast probable trajectories accurately, even several years in advance.

For illustration in Fig. 3, we display the prediction results of MagmaClust and Jenss-Bayley for one random individual for 3 prediction ranges (ages 2 to 10 y, 4 to 10 y, and 6 to 10 y). An intuitive way to represent the time-varying uncertainty in the trajectory predicted by MagmaClust is to draw and display multiple trajectories from the posterior distribution, as in Fig. S1 and Fig. 3A. We observed that MagmaClust’s forecasts provided an accurate mean trend for all three different prediction ranges, with additional data seeming to narrow the range of probable trajectories and prediction errors (Fig. 3B). In contrast, we observed that Jenss-Bayley predictions quickly diverged from testing values (Fig. 3C).

### MagmaClust based tool to predict overweight/obesity in childhood using history of BMI growth

We leveraged MagmaClust’s BMI forecasts to assess the risk of overweight/obesity at age 10 y using BMI data observed up to different age thresholds. To identify overweight/obesity at age 10 y, we used the sex-specific 90th percentile from BMI reference data obtained from Singaporean children (*BMI* > 22 for girls and *BMI* > 22.8 for boys) [7]. Note that while we illustrate the use of the tool for predicting overweight/obesity status at age 10 y, the tool can be readily adapted for different weight thresholds and different ages. For each child, we generated 100,000 instantiations of probable trajectories from the predictive posterior distribution provided by MagmaClust to count the proportion of trajectories that crossed the overweight threshold at the age of 10 y. We represent in Fig. 4 an example of a random boy (A) and girl (B). In both cases, for the purpose of visualization, we display 100 probable trajectories (out of 100,000 probable trajectories of the same individual) predicted from ages 0 to 6 y and focus on the curves crossing the sex-specific overweight/obesity threshold. The ratio of the number of trajectory instantiations of a child crossing overweight threshold to the total number of trajectory instantiations of a child was defined as the probability of overweight/obesity for a given child. Although the mean trend of those predictions is below the overweight/obesity threshold, the probability of being overweight at age 10 y remains non-null (4% for the boy and 3% for the girl). Such a tool provides a valuable risk quantification of undesirable events several years in advance by leveraging the well-calibrated uncertainty coming from MagmaClust results.

To evaluate the practical utility of our overweight probabilities, we compared the observed and predicted overweight status at age 10 y, using BMI data below different age thresholds (2 y, 4 y, 6 y, & 8 y). Within the 577 individuals in the test set, only 297 (148 girls, 149 boys) had BMI data at age 10. Among them, 40 children crossed overweight cutoff at age 10 y. In Fig. 5A, we show the computed probabilities of overweight/obesity at age 10 y for all 297 children. We notice that as we increased the age range of training data for forecasting, the accuracy of identification of true children with overweight/obesity greatly improved. These probabilities provide a continuous degree of risk of overweight/obesity. To evaluate the match between these overweight/obesity probabilities and observed weight status at age 10 y, we considered 5% probability as a decision cutoff. The corresponding sensitivity, specificity, accuracy, positive predictive value (PPV) and negative predictive value (NPV) for different prediction ranges are shown in Fig. 5B. The sensitivity greatly increased with the increase in number of observed timepoints used for trajectory prediction. When the trajectories were predicted using data till age 8 y, 90% of children who had overweight/obesity at age 10 y were correctly identified. The positive predictive value remained moderate, increasing from 0.44 to 0.71 for prediction intervals ranging from 8 y to 2 y. Overall, MagmaClust attributed low probabilities to the vast majority of children who turned out not to be overweight at age 10. The specificity of the method for detecting overweight/obesity status at age 10 remained very high (0.94 to 0.96) even for predictions starting at age 2 y. Concordantly, the negative predictive value was also consistently high (0.80 to 0.98) over the different prediction intervals. We observed that in some cases, the predicted trajectories using BMI data up to a certain age cutoff deviate significantly from the observed trajectories, with the predictions recalibrating towards the observed trajectories when data from addition timepoints are included in the model (Fig. S2).

## Discussion

We developed and evaluated a non-parametric and probabilistic framework for BMI trajectory modeling and forecasting individual childhood BMI trajectories, and forecasting the risk of childhood overweight/obesity status using MagmaClust. While MagmaClust identifies cluster-specific mean processes as an intermediate step of its prediction pipeline, these cluster-specific mean trends already constitute meaningful insights into distinctive BMI childhood growth patterns in this population. One critical aspect to note is that, as in other mixture models, an individual child may not strictly belong to one cluster. Instead, each cluster’s weight for a child corresponds to the membership probability of belonging to that cluster. As the sum of all weights equals 1, we assume that each child belongs to a mixture of clusters. The estimates of BMI trajectories from MagmaClust were compared to two existing methods: cubic B-splines with fixed effects only and Jenss-Bayley model accounting for random effects. We found that both MagmaClust and Jenss-Bayley models remained robust regardless of the missing values proportions, as these methods account for random effects in the cohort. However, it was observed that before age 2 y, when BMI changed rapidly, MagmaClust accurately captured the peak region, resulting in smaller MSE. Also, as the proportion of missing values increased from 10% to 90%, MSE increased from 0.90 to 2.84 in the case of MagmaClust, compared with a 0.94 to 8.06 increase for Jenss-Bayley. Regarding BMI forecasting into the future, only MagmaClust demonstrated accurate predictions up to age 10 y. The accuracy of BMI predictions remained consistent across various intervals for the forecasting periods, ranging from ages 2–10 y to ages 8–10 y. The ability to visualize the uncertainty of the predictions by drawing multiple curves from the posterior distribution is another major advantage of probabilistic frameworks compared to frequentist approaches like Cubic B-splines and Jenss-Bayley. Cubic B-splines were found to be not robust to missing values for retrospective modeling and not very useful for prospective forecasting. Splines are known to be particularly sensitive to data sparsity on boundaries where they tend to provide inaccurate linear extrapolations [16]. One important point regarding mixed-effect Jenss-Bayley and MagmaClust is that borrowing information from the entire population may have the effect of pulling more extreme trajectories towards the mean trajectory (or cluster-specific mean trajectory in MagmaClust). This phenomenon (referred to as shrinkage in mixed-effect models) may have some beneficial effects in real-world settings. Compared to a prospective longitudinal cohort study, real-world longitudinal BMI growth data is likely to be quite noisy (due to measurement errors, varying instruments, non-standardized measurement protocols and outliers) as well as sparse (due to irregular sampling and missing height/weight values). Against this background, the mixed-effect Jenss-Bayley model and MagmaClust may produce more robust trajectory estimates, given their ability to borrow growth information from across the population when compared to methods that only use growth data from a single individual.

As an additional downstream analysis for quantifying the uncertainty of predicted trajectories, we computed instantiations of BMI predictions (up to 100,000 possible trajectories) for each individual child. This allowed us to compute, at any age, the probability of crossing the overweight thresholds. Such an approach provides a practical tool to assess the probability of acquiring overweight/obesity in the future from historical BMI growth data. Note that the proposed approach makes efficient use of the complete longitudinal BMI growth trajectory measured up to a certain time point for predicting future risk of overweight/obesity. This is in contrast to earlier approaches which have usually relied on continuous weight/BMI or categorical weight status at a single time point [25–29], change in weight/BMI *z*-scores between two time points [28, 30], or timing/intensity of growth milestones like infancy BMI peak [31, 32] or adiposity rebound [33, 34] for predicting future overweight/obesity risk. In the current work, for identifying overweight/obesity status at age 10 y, we used an arbitrary decision cutoff of 5% for the proportion of predicted trajectories crossing the overweight threshold at age 10 y. This cutoff could easily be adapted depending on the clinical context and needs (e.g. a higher cutoff can be used for triaging only high-risk children towards more intensive interventions). From empirical evaluation, we reported high accuracy at all ages (86% to 94%) for this prospective overweight/obesity status detection procedure and a quickly increasing sensitivity, allowing us to identify 20% of the actual overweight children using BMI data from age 0 to 2 y, rising up to 90% when using data from ages 0 to 8 y. The PPV increased from 44% to 71% in the same interval. The specificity and NPV for detecting overweight/obesity at age 10 y were consistently high for all prediction intervals (over 94% and 80% respectively). The proposed MagmaClust-based approach for probabilistic prediction of future obesity risk only uses prior BMI growth history as input. Note that, while we have not explicitly used conventional risk factors for obesity like short breastfeeding duration, early time of weaning, low physical/high sedentary activity and diet quality for the prediction of obesity risk, these factors interact with the genetic growth potential to influence the BMI growth trajectory. Obesogenic risk factors cannot cause obesity without first modifying the BMI growth trajectories. Thus, using BMI growth history to predict future obesity risk implicitly also takes prior exposure to environmental risk factors also into account.

In this study, we have cut off BMI growth data at arbitrary ages (2 y, 4 y, 6 y, & 8 y) to predict future overweight/obesity. For clinical translation, these times could be redefined based on visits to pediatricians (e.g. well-child visits and vaccination visits). If previously forecasted mean BMI trajectories for a child are available, then marked deviations from previously forecasted trajectories may be a result of adverse environmental or behavioral factors (e.g. sudden changes in nutritional patterns or physical activity) and may provide a signal for pediatricians to prioritize these children for follow-ups and interventions.

While existing literature emphasizes the significance of monitoring and detailing the longitudinal growth of children to gain a more nuanced understanding of potential predispositions for adverse adult health [35–38], we introduced a methodology to prospectively predict future BMI growth trends. MagmaClust possesses the flexibility to undergo retraining by incorporating future growth data, for instance, for capturing post-pubertal growth trends or incorporating growth data from more geographies and ethnicities to generate more representative models. This adaptability enables the extension of predictions to older ages, providing a robust tool for ongoing assessments of childhood growth trajectories. This offers both a methodological advancement and a practical tool to monitor expected growth trends and possible deviations from expected trends during childhood and can enable appropriate interventions.

## Supplementary information

Supplementary Material

## Data Availability

The data used in the manuscript are available on request, on approval by the GUSTO executive committee. Data catalog and request form can be found at https://gustodatavault.sg/.

## Code availability

The MagmaClust framework is implemented as an R package called MagmaClust, available on the CRAN, while a development version can be found on GitHub (https://github.com/ArthurLeroy/MagmaClustR). To help experiments’ reproducibility, all computations, results and trained models presented in this article are stored in the following GitHub repository https://github.com/ArthurLeroy/BMI_MagmaClust.
