# Correction: Prospective prediction of childhood body mass index trajectories using multi-task Gaussian processes

**DOI:** 10.1038/s41366-024-01699-w

**Published:** 2024-12-29

**Authors:** Arthur Leroy, Varsha Gupta, Mya Thway Tint, Delicia Shu Qin Ooi, Fabian Yap, Ngee Lek, Keith M. Godfrey, Yap Seng Chong, Yung Seng Lee, Johan G. Eriksson, Mauricio A. Álvarez, Navin Michael, Dennis Wang

**Affiliations:** 1 Department of Computer Science, The University of Manchester, Manchester, UK; 2 Institute for Human Development and Potential, Agency for Science Technology and Research (A*STAR), Singapore, Republic of Singapore; 3 Bioinformatics Institute, Agency for Science Technology and Research (A*STAR), Singapore, Republic of Singapore; 4 Department of Paediatrics, Yong Loo Lin School of Medicine, National University of Singapore, Singapore, Republic of Singapore; 5 Department of Paediatrics, KK Women’s and Children’s Hospital, Singapore, Republic of Singapore; 6 Duke-NUS Medical School, Singapore, Republic of Singapore; 7 MRC Lifecourse Epidemiology Centre and NIHR Southampton Biomedical Research Centre, University of Southampton and University Hospital Southampton NHS Foundation Trust, Southampton, UK; 8 Department of Obstetrics and Gynaecology and Human Potential Translational Research Programme, Yong Loo Lin School of Medicine, National University of Singapore, Singapore, Republic of Singapore; 9 Division of Paediatric Endocrinology, Department of Paediatrics, Khoo Teck Puat-National University Children’s Medical Institute, National University Hospital, National University Health System, Singapore, Republic of Singapore; 10 Department of General Practice and Primary Health Care, University of Helsinki, Helsinki, Finland; 11 Folkhälsan Research Center, Helsinki, Finland; 12 Department of Computer Science, University of Sheffield, Sheffield, UK; 13 National Heart and Lung Institute, Imperial College London, London, UK

**Keywords:** Obesity, Paediatrics

Correction to: *International Journal of Obesity* 10.1038/s41366-024-01679-0, published online 15 November 2024

In the original article, a previous version of figure 5B was inadvertently published. The information in the ‘Results and Discussion’ section as well as in the caption are not affected by this correction.

Figure 5 in the original article:
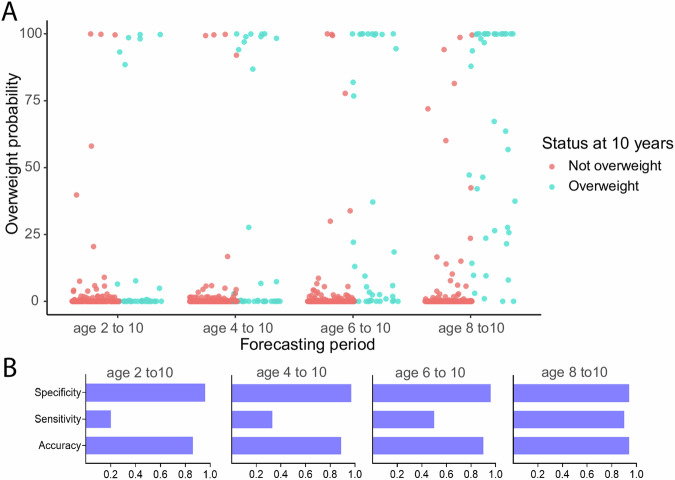


Corrected figure 5:
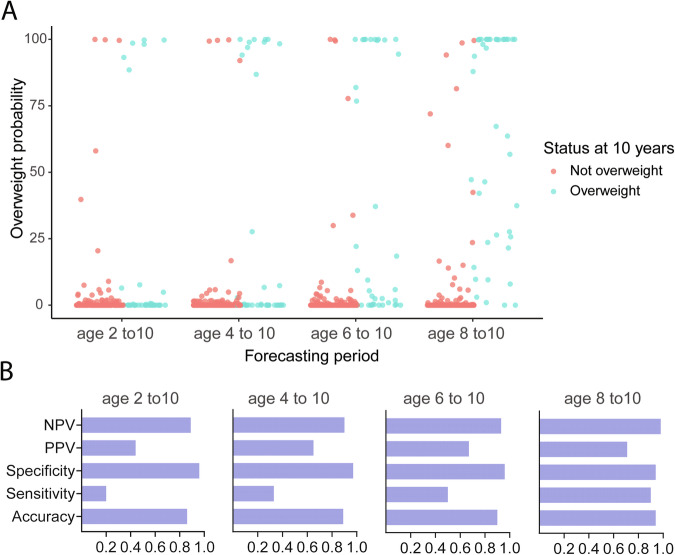


The original article has been corrected.

