# Supplementary material for: Prospective prediction of childhood body mass index trajectories using multi-task Gaussian processes
Source: Int J Obes (Lond). 2024 Nov 15;49(2):340–7. doi: 10.1038/s41366-024-01679-0 (PMC11805709; doi:10.1038/s41366-024-01679-0)
Supplement: Supplementary file 1 — Supplementary Material [file 41366_2024_1679_MOESM1_ESM.docx]

**SUPPLEMENTARY MATERIALS**


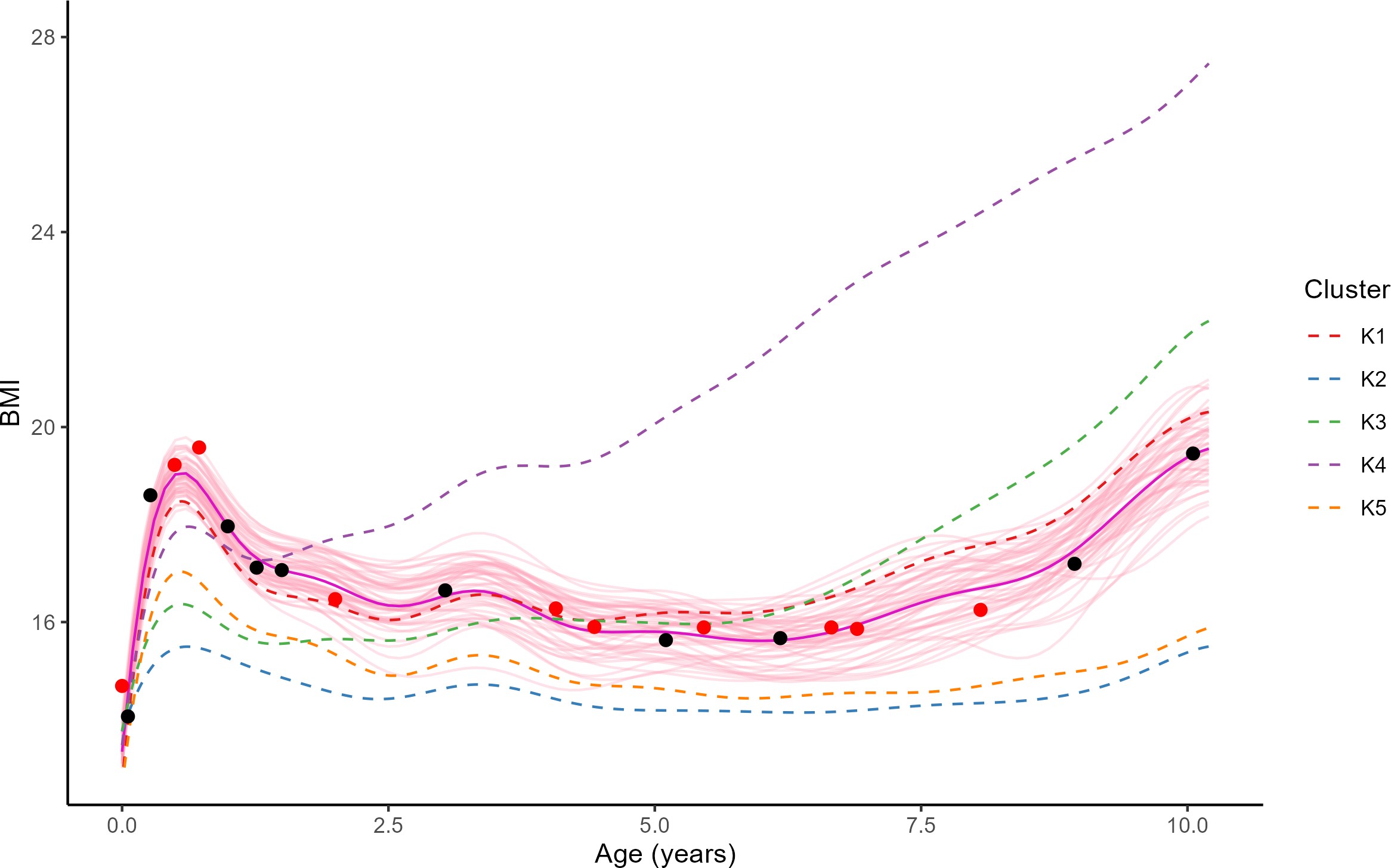

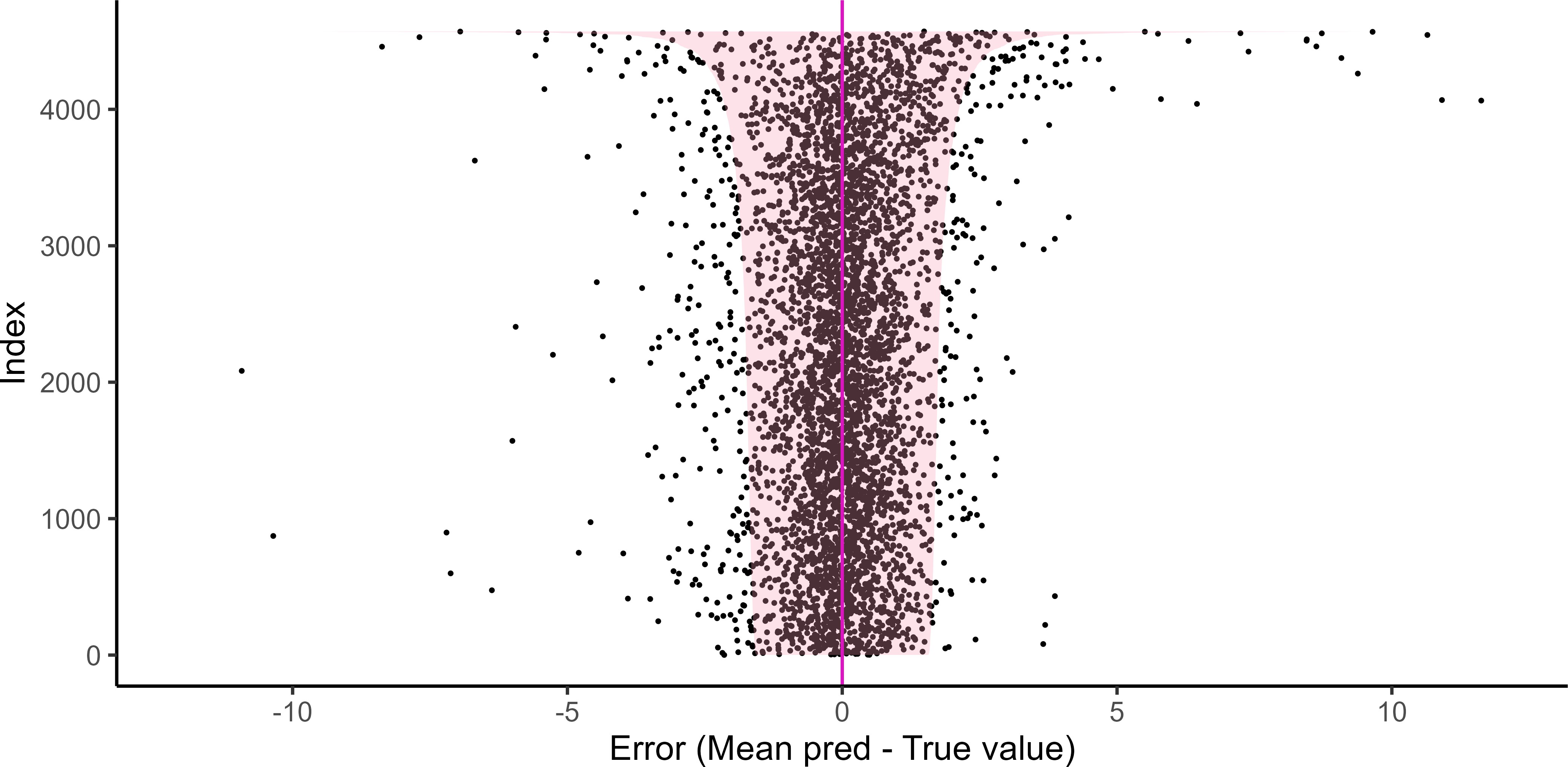


A

B

Figure S1 (A) depicts the predicted curve for a random individual with 50% missing data obtained by the 5-cluster MagmaClust model overlaid on the cluster-specific mean curves. (B) the error between predicted and observed BMI values, sorted by increasing variance.


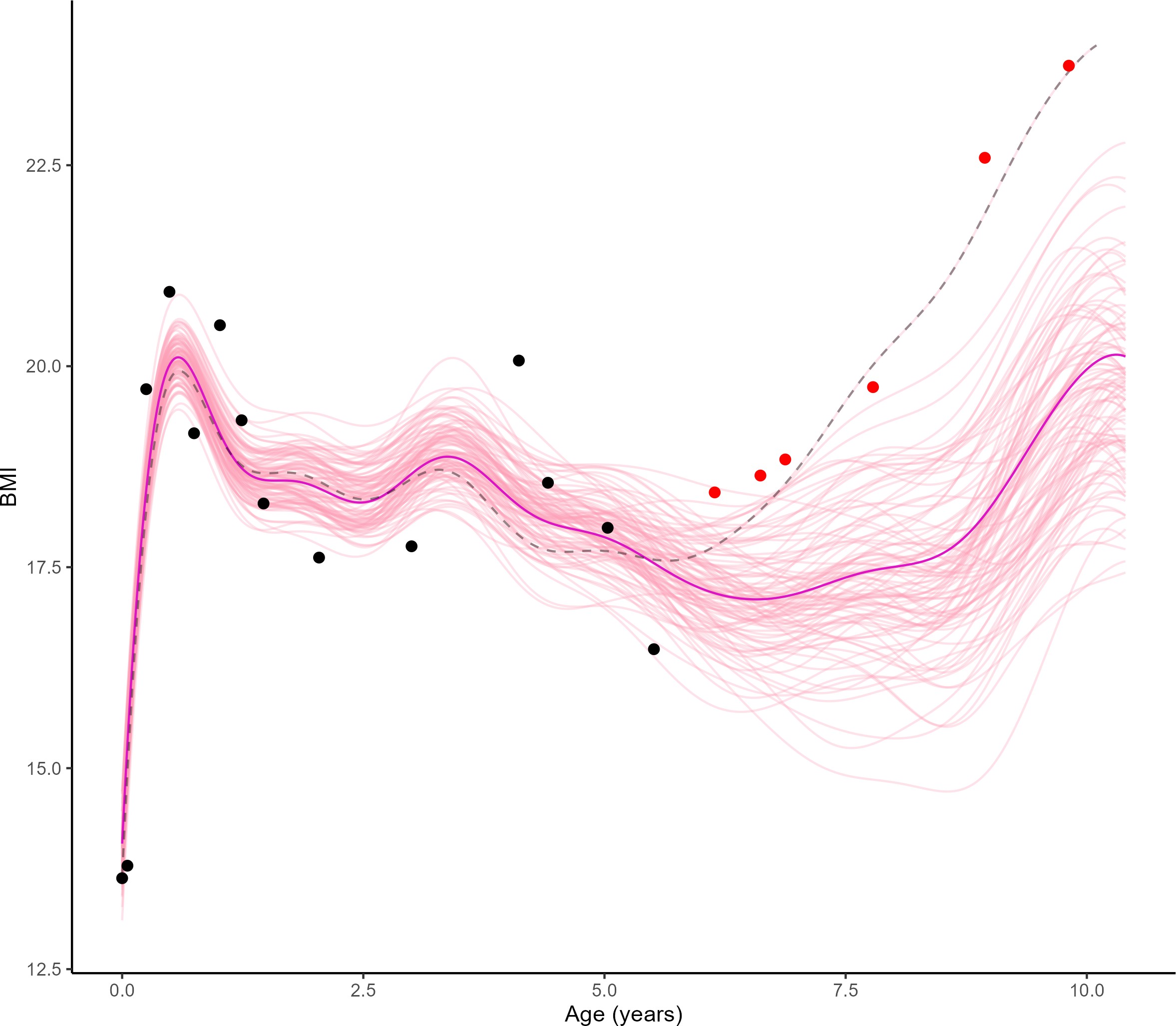

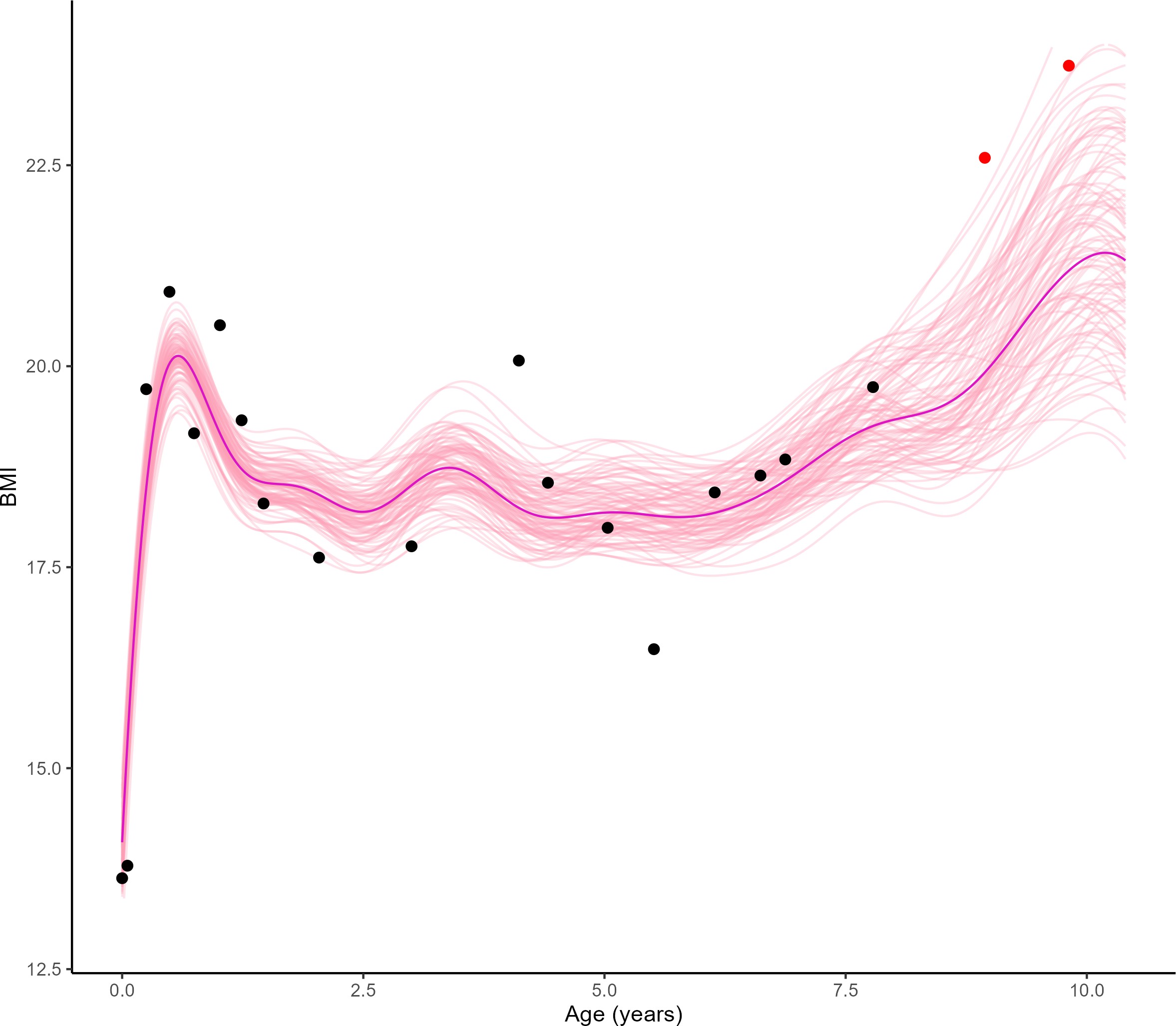


A

B

Fig. S2: (A) Example of deviation of observed BMI from MagmaClust predictions. Posterior sample trajectories (pink curves) are represented around the mean trend (purple curve) based on observed growth until 6 years (black dots), and the actual BMI (red dots) corresponds to low-probability trajectories. (B) When increasing the observation range to 8 years (right panel), expected mean trajectory adapted accordingly, but still deviates from the observed BMI. Deviation from the expected growth trajectory can potentially constitute an alert for clinicians.

Table S1: Number of children with observed weight, height and BMI at each time point. Two children did not have weight and height information at birth but had this information at other instants. Therefore, the total number of children for whom BMI trajectories were available was 1177.

| Age (in month) | Weight | Height | BMI |
| --- | --- | --- | --- |
| 0 | 1179 | 1175 | 1175 |
| 0.75 | 1038 | 1036 | 1035 |
| 3 | 1024 | 1024 | 1024 |
| 6 | 980 | 984 | 980 |
| 9 | 941 | 942 | 941 |
| 12 | 960 | 960 | 958 |
| 15 | 962 | 941 | 940 |
| 18 | 911 | 862 | 857 |
| 24 | 925 | 892 | 891 |
| 36 | 932 | 928 | 925 |
| 48 | 861 | 858 | 858 |
| 54 | 896 | 897 | 895 |
| 60 | 873 | 873 | 872 |
| 66 | 862 | 863 | 862 |
| 72 | 832 | 830 | 830 |
| 78 | 821 | 821 | 821 |
| 84 | 863 | 864 | 863 |
| 96 | 807 | 807 | 807 |
| 108 | 755 | 756 | 755 |
| 120 | 661 | 661 | 661 |

Table S2: Average (sd) values of MSE, *WCIC*_95_ in missing data reconstruction for 577 testing individuals when applying MagmaClust for different numbers of clusters, Jenss-Bayley, and B-splines.

|  | MSE | *WCIC*_95_ |
| --- | --- | --- |
| MagmaClust 2 clusters | 1.55 (5.88) | 92.58 (11.23) |
| MagmaClust 3 clusters | 1.68 (6.93) | 90.92 (12.16) |
| MagmaClust 4 clusters | 1.64 (6.79) | 92.56 (11.69) |
| MagmaClust 5 clusters | 1.69 (6.47) | 91.07 (12.22) |
| Jenss-Bayley | 2.22 (4.31) | Not applicable |
| Splines | 8.11 (258.17) | Not applicable |

Table S3: Average (sd) values of MSE, *WCIC*_95_ in missing data reconstruction with an increasing percentage of missing data, for 577 individuals when applying MagmaClust with 5 clusters, Jenss-Bayley, and B-splines

|  | MagmaClust  MSE *WCIC*_95_ | Jenss-Bayley  MSE | Splines  MSE Failed computations |
| --- | --- | --- | --- |
| Missing data ratio |  |  |  |
| 10% | 0.90 (2.36) 90.90 (28.8) | 0.94 (1.99) | 1.86 (4.78) 3.1% |
| 25% | 1.39 (3.17) 91.60 (15.84) | 1.55 (1.96) | 2.60 (8.61) 2.5% |
| 50% | 1.71 (3.16) 91.38 (12.64) | 2.63(2.98) | 3.57 (21.5) 5.2% |
| 75% | 2.00 (2.97) 93.00 (10.84) | 3.29(4.44) | 5.14 (15.5) 68.5% |
| 90% | 2.84 (8.74) 95.06 (9.26) | 8.06 (9.49) | / 100% |

Table S4: Average (sd) values of MSE and *WCIC*_95_ in forecasting using an increasing number of observed early data points for 577 testing individuals when applying MagmaClust with 5 clusters, Jenss-Bayley, and B-splines.

|  | MagmaClust MSE *WCIC*_95_ | Jenss-Bayley  MSE | Splines  MSE |
| --- | --- | --- | --- |
| Forecasting |  |  |  |
| from 2 to 10 years | 4.11 (12.77) 95.21 (21.361) | 13.39(22.57) | 352.47 (1386.57) |
| from 3 to 10 years | 3.94 (12.65) 94.55 (22.71) | 12.31(19.03) | 156.73 (409.61) |
| from 4 to 10 years | 3.11 (8.83) 95.11 (21.58) | 8.69(13.27) | 52.10 (157.69) |
| from 5 to 10 years | 2.81 (8.60) 94.47 (22.85) | 5.46(8.37) | 26.10 (95.26) |
| from 6 to 10 years | 2.55 (7.96) 94.46 (22.88) | 4.18(7.59) | 23.06 (88.73) |

**MagmaClust versus conventional growth modelling approaches**

Broadly, two classes of methods have been used for characterizing longitudinal childhood BMI trends – group-based trajectory modelling (GBTM) and individual trajectory modelling. GBTM approaches like latent class growth analysis (LCGA) and latent class growth mixture modelling (LCGMM) have been used to identify distinct clusters of longitudinal growth trajectories, such that children within a cluster have relatively homogeneous growth trajectories^1-3^. Grouping children based on distinctive growth patterns has been motivated by the fact that children within a cluster may share similar underlying drivers as well as future health outcomes. Such methods provide a probability of belonging to a growth cluster for each subject, and the mean trajectories within each cluster describe the distinctive growth patterns. However, such models have several limitations. Such techniques have usually been used to model relatively simple trajectory patterns (e.g. linear or quadratic trends) and face a lot of convergence issues with more complicated patterns^4^. Hence, they may not capture all possible biological trajectory patterns that exist in the population. They have been commonly used for modelling age- and sex-standardized growth metrics, which have less complex trajectory shapes than non-standardized growth metrics.

GBTM-style approaches have been less commonly used for modelling individual growth trajectories. This has been usually performed using parametric models or nonparametric models. Parametric models use specific mathematical functions that leverage prior knowledge of the expected childhood growth trends and have biologically interpretable parameters. A key limitation of such approaches is that while there are simple parametric forms that can model weight or height (e.g. Jenss-Bayley^5^ and Reed^6^ models), it is difficult to capture the complex dynamics of childhood BMI in a simple parametric form. Instead, weight and height have to be modelled separately, which can then be used to estimate the BMI curve^7^. Hence, errors in the individual weight and height models can propagate to the estimated BMI model. Alternatively, BMI can be directly modelled using flexible functions that can model arbitrary shapes like fractional polynomials and splines^8^. In these models, the estimated parameters have no biological meaning. However, such approaches are optimised for interpolating between observed growth measurements and may have poor performance extrapolating growth trends outside the observed window.

In the current work, we proposed to use a recent multi-task Gaussian processes algorithm called MagmaClust ^9^ , which has been previously used for time series forecasting for growth modelling. In contrast to the previous growth models, Gaussian processes-based methods offer a probabilistic non-parametric framework by defining a prior distribution over functions, allowing us to capture complex non-linear relationships while accounting for uncertainty. MagmaClust performs functional curve clustering as well as prediction of individual trajectories within the same model, obviating the need for separate approaches for growth clustering and individual trajectory modelling. Thus, it represents an advancement over traditional GBTM and individual trajectory modelling approaches.

Formally, a Gaussian process (GP) is a random process over functions (or curves) that is characterized by a specific mean and covariance function. Intuitively, GPs generalize traditional multivariate Gaussian distributions as any evaluation of a GP at a finite number of points is a multivariate Gaussian, parameterized by the corresponding mean vector and covariance matrix (see the monograph^10^ for detailed explanations). Observed individual growth trajectories can be visualized as specific instantiations of different Gaussian processes. A naïve approach would be to model each individual trajectory using separate, independent GPs. However, this ignores the structure that exists in the data since the individual trajectories all represent the same underlying growth process and the potential for improving learning by sharing information across the different individual trajectories.

In an earlier iteration of the framework (called Magma^11^), this information sharing was achieved by expressing the trajectory of each individual as a sum of a common mean GP shared by all individuals, and an individual-specific GP. MagmaClust advances this approach by allowing the simultaneous clustering of growth patterns and defining a common mean GP for each cluster specifically. Similar to LCGMM, MagmaClust is a mixture model that returns the membership probability of different clusters for each individual (individuals can have non-zero probabilities of belonging to multiple clusters). Moreover, the final prediction of an individual growth curve is expressed as a GP mixture of all cluster-specific predictions, weighted by the adequate membership probabilities. The sharing of longitudinal growth information across multiple individuals and the allowance for clustering of growth trajectories offer more accurate individual predictions while accounting for uncertainty thanks to the probabilistic nature of GPs. The method can accommodate arbitrary trajectory shapes, naturally deals with irregular measurements, and has been designed to provide robust predictions even with missing values.

In the present paper, we used the R package MagmaClustR, providing an up-to-date implementation of the methods described. As for any GP-based methods, practitioners can choose a dedicated kernel to incorporate prior knowledge on the signal to capture. A somewhat classical choice when modelling smooth functions is the squared exponentiated (also called RBF) kernel, which proved to be well-adapted in our experiments. This kernel is characterized by two hyper-parameters, namely the *variance* and the *lengthscale*, optimized during the learning procedure (through an EM algorithm) of MagmaClust. Those hyper-paramaters are computed for each cluster and each individual, and thus can be analyzed to provide information on the properties of individual trajectories and subsequent predictions. Similarly, the clustering procedure within MagmaClust relies on a mixture of GPs, which also leverages parameters being optimized: the clusters’ membership probabilities. In such probabilistic frameworks, each individual has a specific probability of belonging to each cluster. Therefore, analyzing the posterior values of those parameters can also provide valuable insights to clinicians in understanding the particularities of individual trajectories.

**Cubic B-splines (fixed effects only)**

We also conducted the experiments using a standard splines approach as a baseline. More specifically, we defined our BMI trajectories as a decomposition of cubic B-splines^12,13^. Each child was treated individually by fitting an independent B-spline decomposition on its data points. The smoothing computations were performed thanks to the *smooth.spline* function of the *stat* R package. While utilizing cubic B-splines is a standard choice to obtain a smooth and flexible fit for functions, it induces a minimal requirement of 4 data points to be computed. Therefore, some experiments involving missing data could lead to numerical errors when the amount of observed data was too low for an individual. We specifically referred to those cases as *failed computations* in Table S3.

**Parametric Jenss-Bayley method with random effects**

The Jenss-Bayley parametric growth model was originally proposed for modelling weight and height between birth and 6-8 years^5^. We used a modified and parameterized form of the Jenss-Bayley model^14,15^ for modelling both weight and height/length that includes an additional quadratic term that can account for growth during puberty and is suitable for modelling growth up to age 12 years. The modified Jenss-Bayley weight and height models were hierarchically fitted with a non-linear mixed effect model using the *saemix* package in R. Children with at least two weight and height measurements were included in the analysis. The individually fitted weight and height trajectories were subsequently used for calculating the BMI at different time points.

**Evaluation metrics**

For clarity, let us recall that *N* denotes the number of individuals, *T_i_* the number of time points observed for the *i*-th individual, and *K* is the number of clusters, whereas *y_obs_* and *y_pred_* represent the functions of observed and predicted BMI, respectively. Formally, we define the Mean Squared Error (MSE) in the subsequent experiments as follows:


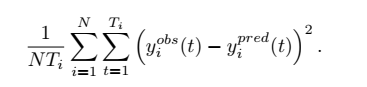


Moreover, an additional measure of uncertainty quantification, introduced earlier^9^, is used to evaluate whether the observations belong to the predicted credible intervals as expected. Namely, the weighted *CI*_95_ coverage (*WCIC*_95_) is defined as:


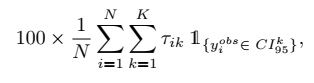


Where ${CI}_{95}^{k}$ represents 95% credible interval computed for the *k*-th cluster, and *τ_ik_* corresponds to the probability for the *i*-th individual to belong to the *k*-th cluster. When interpreting this metric, the closer to the theoretical value of 95%, the better.

**Supplementary References**

1 Jung, T. & Wickrama, K. An introduction to latent class growth analysis and growth mixture modeling. *Social and personality psychology compass* **2**, 302-317 (2008).

2 Nylund, K. L., Asparouhov, T. & Muthén, B. O. Deciding on the Number of Classes in Latent Class Analysis and Growth Mixture Modeling: A Monte Carlo Simulation Study. *Structural Equation Modeling: A Multidisciplinary Journal* **14**, 535-569, doi:10.1080/10705510701575396 (2007).

3 Mattsson, M. *et al.* Group-based trajectory modelling for BMI trajectories in childhood: A systematic review. *Obes Rev* **20**, 998-1015, doi:10.1111/obr.12842 (2019).

4 McNeish, D. & Harring, J. R. Improving convergence in growth mixture models without covariance structure constraints. *Stat Methods Med Res* **30**, 994-1012, doi:10.1177/0962280220981747 (2021).

5 Jenss, R. M. & Bayley, N. A mathematical method for studying the growth of a child. *Human Biology* **9**, 556 (1937).

6 Front Matter. *Human Biology* **9** (1937).

7 Carles, S. *et al.* A Novel Method to Describe Early Offspring Body Mass Index (BMI) Trajectories and to Study Its Determinants. *PLoS One* **11**, e0157766, doi:10.1371/journal.pone.0157766 (2016).

8 Tilling, K., Macdonald-Wallis, C., Lawlor, D. A., Hughes, R. A. & Howe, L. D. Modelling childhood growth using fractional polynomials and linear splines. *Ann Nutr Metab* **65**, 129-138, doi:10.1159/000362695 (2014).

9 Leroy, A., Latouche, P. MagmaClustR: Clustering and Prediction using Multi-Task Gaussian Processes with Common Mean. [*https://arthurleroy.github.io/MagmaClustR/*](https://arthurleroy.github.io/MagmaClustR/) (2023).

10 Rasmussen, C. E. & Williams, C. K. I. (The MIT Press, 2005).

11 Leroy, A., Latouche, P., Guedj, B. & Gey, S. MAGMA: inference and prediction using multi-task Gaussian processes with common mean. *Machine Learning* **111**, 1821-1849, doi:10.1007/s10994-022-06172-1 (2022).

12 de Boor, C. On calculating with B-splines. *Journal of Approximation Theory* **6**, 50-62, doi:<https://doi.org/10.1016/0021-9045(72)90080-9> (1972).

13 Boor, C. d. in *Applied Mathematical Sciences.*

14 Botton, J., Scherdel, P., Regnault, N., Heude, B. & Charles, M. A. Postnatal weight and height growth modeling and prediction of body mass index as a function of time for the study of growth determinants. *Ann Nutr Metab* **65**, 156-166, doi:10.1159/000362203 (2014).

15 Botton, J., Heude, B., Maccario, J., Ducimetière, P. & Charles, M. A. Postnatal weight and height growth velocities at different ages between birth and 5 y and body composition in adolescent boys and girls. *Am J Clin Nutr* **87**, 1760-1768, doi:10.1093/ajcn/87.6.1760 (2008).
